# Why soft contacts are stickier when breaking than when making them

**DOI:** 10.1126/sciadv.adl1277

**Published:** 2024-03-06

**Authors:** Antoine Sanner, Nityanshu Kumar, Ali Dhinojwala, Tevis D. B. Jacobs, Lars Pastewka

**Affiliations:** ^1^ Department of Microsystems Engineering (IMTEK), University of Freiburg, Georges-Köhler-Allee 103, 79110 Freiburg, Germany.; ^2^ Cluster of Excellence *liv*MatS, Freiburg Center for Interactive Materials and Bioinspired Technologies, University of Freiburg, Georges-Köhler-Allee 105, 79110 Freiburg, Germany.; ^3^ Institute for Building Materials, ETH Zurich, Zurich 8093, Switzerland.; ^4^ School of Polymer Science and Polymer Engineering, The University of Akron, Akron, OH 44325, USA.; ^5^ Science & Technology Division, Corning Incorporated, Corning, NY 14831, USA.; ^6^ Department of Mechanical Engineering and Materials Science, University of Pittsburgh, 3700 O’Hara Street, Pittsburgh, PA 15261, USA.

## Abstract

Soft solids are sticky. They attract each other and spontaneously form a large area of contact. Their force of attraction is higher when separating than when forming contact, a phenomenon known as adhesion hysteresis. The common explanation for this hysteresis is viscoelastic energy dissipation or contact aging. Here, we use experiments and simulations to show that it emerges even for perfectly elastic solids. Pinning by surface roughness triggers the stick-slip motion of the contact line, dissipating energy. We derive a simple and general parameter-free equation that quantitatively describes contact formation in the presence of roughness. Our results highlight the crucial role of surface roughness and present a fundamental shift in our understanding of soft adhesion.

## Teaser

Surface roughness pins the perimeter of adhesive contacts, leading to a hysteresis when joining and separating soft solids.

## INTRODUCTION

**Fig. 1.**
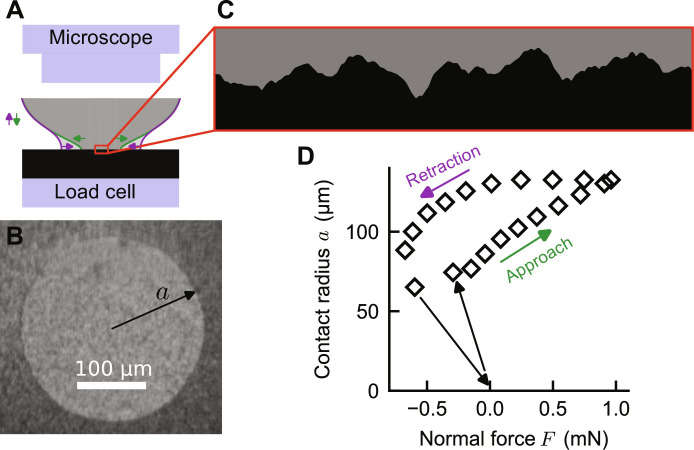
Phenomenology of adhesive contact. (**A**) Many contacts can be described as spheres making contact with a flat surface. For soft materials, microscopic interactions are strong enough that the solids deform substantially near the contact edge. The darker gray region illustrates the contact during approach and the lighter gray region the contact at the same rigid-body penetration during retraction, indicating a hysteresis between approach and retraction. (**B**) The contact forms a circle for contacting spheres, and its radius *a* can be measured from in situ optical images of the contact area. (**C**) Most natural and technical surfaces are rough so the solid needs to elastically deform to come into conforming contact. (**D**) The contact radius is larger and the normal force is more adhesive (negative) during retraction than during approach, as is also shown schematically in (A). The pull-off force is the most negative force on these curves.

Insects, pick-and-place manufacturing, engineered adhesives, and soft robots use soft materials to stick to surfaces even in the presence of roughness. These materials stick to each other because of attractive van der Waals or capillary interactions at small scales (*1*). The strength of these interactions is commonly described by the intrinsic work of adhesion *w*_int_, the energy that is gained by these interactions per surface area of intimate contact. This work of adhesion is most typically measured from the pull-off force *F*_pulloff_ = −3π*w*_int_*R*/2 of a soft spherical probe (see Fig. 1A) with radius *R* which makes a circular contact with radius *a* (see Fig. 1B) (*2*). For hard solids, the measured apparent work of adhesion is smaller than the intrinsic value *w*_int_ because roughness limits the area of intimate contact to the highest protrusions (*3*, *4*). In contrast, soft solids are sticky because they can deform to come into contact with a large portion of the rough topography. The overall strength of the adhesive joint is then determined by the balance of the energy gained by making contact and the elastic energy spent in conforming to the surface. Following Persson and Tosatti (*5*), energy conservation implies that surface roughness reduces the apparent work of adhesion to

$$w_{\text{PT}}=w_{\text{int}}-e_{\text{el}}$$ (1)

where *e*_el_ is the elastic energy per unit contact area required to conform to the roughness (Fig. 1C). As shown in Fig. 1D, experiments typically follow different paths during approach and retraction, leading to different apparent values for work of adhesion for making and breaking contact, *w*_appr_ and *w*_retr_. This adhesion hysteresis (*6*, *7*) contradicts Persson and Tosatti’s balance of energy, which gives the same value *w*_PT_ for approach and retraction. The common explanation for this hysteresis is either contact aging or viscoelasticity (*1*, *8*).

In this article, we show that adhesion hysteresis emerges even for perfectly elastic contacts and in the absence of contact aging and viscoelasticity because of surface roughness. We present a crack perturbation model (*9*–*11*) and experimental observations that reveal discrete jumps of the contact perimeter. These stick-slip instabilities are triggered by local differences in fracture energy between roughness peaks and valleys. Pinning of the contact perimeter (*12*–*14*) retards both its advancement when coming into contact and its retraction when pulling away. Our model quantitatively reproduces the hysteresis observed in experiments and allows us to derive analytical predictions for its magnitude, accounting for realistic rough geometries across orders of magnitude in length scale (*15*, *16*). For soft spherical probes, we can describe the circular contact perimeter as a crack. The crack front is in equilibrium when Griffith’s criterion is fulfilled (*17*): The energy per unit area required locally for opening the crack, the fracture energy *w*_loc_, is equal to the energy released from the elastic deformation, *G*δ*A* = *w*_loc_δ*A*, where δ*A* is the contact area swept out by the moving crack front. A more common way of writing this equation is

$$G=w_{\text{loc}}$$ (2)

where both the elastic energy release rate *G* and the fracture energy *w*_loc_ should be interpreted as forces per unit crack length. Johnson, Kendall, and Roberts (JKR) (*2*) derived the expression for the energy release rate *G* for a smooth spherical indenter, *G* = *G*_JKR_(*b*, *a*). Equation 2 then allows the evaluation of not just the pull-off force, but of all functional dependencies between rigid-body displacement *b*, contact radius *a*, and normal force *F* during contact.

For smooth spheres, the fracture energy is the intrinsic work of adhesion, *w*_loc_ = *w*_int_, which for chemically homogeneous contacts does not vary with position. We will show below that surface roughness can be transformed into a field *w*_loc_(*x*, *y*), which describes the fluctuation of the effective fracture energy in the equivalent smooth contact. Since the process of opening and closing adhesive contacts is locally reversible, the fracture energy *w*_loc_ can be interpreted as an effective local work of adhesion. Equation 2 must then hold independently for each point on the contact perimeter. We start our analysis by assuming that *w*_loc_(*x*, *y*) varies with position and by showing that this is sufficient to yield a hysteresis in the adhesive contact cycle.

## RESULTS

### Axisymmetric chemical heterogeneity

**Fig. 2.**
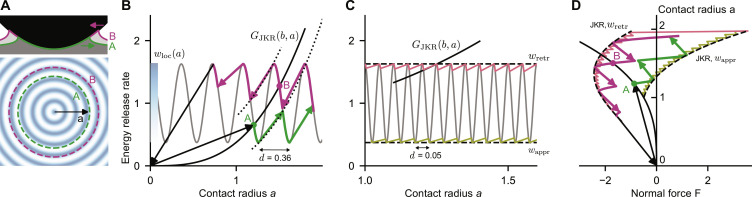
Simplified axisymmetric contact demonstrating the physical origin of the adhesion hysteresis. The indenter is a perfect sphere with axisymmetric heterogeneity in local adhesion *w*_loc_(*a*). (**A**) Cross section of the contact at rigid-body penetration *b* = 0 (top) and top view of the axisymmetric work-of-adhesion heterogeneity *w*_loc_(*a*) (bottom). The blue color indicates regions of high adhesion. (**B**) Elastic energy release rates in an approach-retraction cycle for a sinusoidal work of adhesion *w*_loc_(*a*) with wavelength *d* = 0.36 (gray line). The black line shows the elastic energy release rate *G*_JKR_(*b*, *a*) as a function of contact radius for fixed rigid-body penetration *b* = 0. Fluctuations of *w*_loc_(*a*) lead to several metastable states A and B at fixed *b*. Arrows indicate elastic instabilities where the contact radius jumps between metastable states. (**C**) Energy release rates in an approach-retraction cycle for a work-of-adhesion heterogeneity with smaller wavelength *d* = 0.05. For short wavelengths, the works of adhesion sampled during approach (light green curve) and retraction (light red curve) stay close to the constant values *w*_appr_ and *w*_retr_. (**D**) The contact radius and the normal force during an approach-retraction cycle for wavelength *d* = 0.36 (darker colors) and *d* = 0.05 (lighter colors). The dashed lines are the prediction by the JKR theory using *w*_retr_ and *w*_appr_ for the work of adhesion. The solid black line corresponds to increasing energy release rates at fixed rigid-body penetration *b* = 0. Energy release rates are displayed in units of the average work of adhesion and lengths and forces have been nondimensionalized following the conventions of (*47*, *48*) as described in the Supplementary Materials.

We first demonstrate the physical origin of the adhesion hysteresis using a simplified surface that has concentric rings of high and low adhesion energy, similar to the models by Guduru (*18*), Kesari and Lew (*19*, *20*), and Popov (*21*). Rather than being random, *w*_loc_(*a*) varies in concentric rings of wavelength *d* as a function of distance *a* from the apex of the contacting sphere (Fig. 2A). Figure 2B shows *w*_loc_(*a*) alongside *G*_JKR_(*b*, *a*) for a fixed displacement *b*. Because of the spatial variations of *w*_loc_, there are multiple solutions to Eq. 2 indicated by the labels A and B. Moving into contact from the solution denoted by A leads to an instability where the solution A disappears, at which the contact radius jumps to the next ring of *w*_loc_(*a*). This samples the lower values of *w*_loc_ shown by the green line in Fig. 2B. Conversely, moving out of contact progresses along a different path that samples the higher values of *w*_loc_(*a*), shown by the red line. The combination of fluctuations in *w*_loc_ and the elastic restoring force *G*_JKR_ acts like a ratchet resisting the growing and shrinking of the contact area and leads to a stick-slip motion of the contact line. The line is pinned by the first strong-enough obstacle it encounters, so that it is pinned at a low contact radius when the contact area grows and at a high radius when it shrinks.

In the limit of roughness with a small wavelength, *d* → 0, *G*_JKR_ does not decrease substantially before the contact line arrests at the next peak (see Fig. 2C). In this limit, the contact line samples the minimum values *w*_appr_ of *w*_loc_ during approach and the maximum values *w*_retr_ during retraction. The functional relationship between *b*, *a*, and *F* then becomes identical to the JKR solution for smooth bodies (see equations S4 to S7), but with an apparent work of adhesion that is decreased during approach (*w*_appr_) and increased during retraction (*w*_retr_; see Fig. 2D). In this limit, the hysteresis *w*_retr_ − *w*_appr_ becomes equal to the peak-to-peak amplitude of *w*_loc_(*a*) (*19*).

### Random chemical heterogeneity

**Fig. 3.**
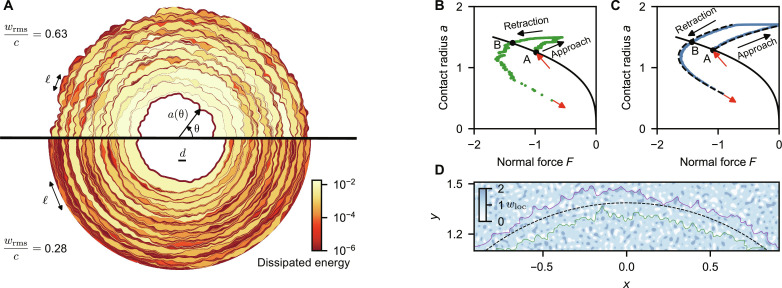
Simulation of crack-front pinning by two-dimensional random heterogeneity. (**A**) Evolution of the contact line during retraction in a crack-front simulation on a two-dimensional random work-of-adhesion field. Each colored patch corresponds to an elastic instability during which the perimeter jumps between two pinned configurations (dark lines), and the color scale represents the energy dissipated during each instability. The Larkin length ℓ corresponds to the smallest extent of these jumps along the perimeter and increases for weaker heterogeneity or for a stiffer line. (**B**) Contact radius as a function of the normal force in the simulation shown at the top of (A). The elastic instabilities lead to sudden jumps in the contact area and in the normal force. The solid black line corresponds to increasing energy release rates at fixed rigid-body penetration *b* = 0 and points A and B show that the contact radius is higher during retraction than during approach. The red arrows show the jump-in and jump-out-of-contact instabilities. (**C**) Contact radius as a function of the normal force in a simulation on a random chemical heterogeneity with a smaller feature size and *w*_rms_/*c* ≈ 0.45. The dashed lines are JKR curves with work of adhesion *w*_appr_ and *w*_retr_ predicted by our theory Eq. 6. (**D**) Contact lines at rigid-body penetration *b* = 0 on the random work-of-adhesion heterogeneity shown by the blue color map. Floppy lines are pinned at higher contact radii during retraction (purple line) than during retraction (green line) because they meander predominantly between regions of low adhesion (white patches) during approach and between regions of high adhesion (dark blue patches) during retraction. In the limit of a rigid line, the perimeter is perfectly circular (dashed line), randomly sampling as many regions of low and high adhesion.

The next step in complexity is moving from a simplified axisymmetric surface to a surface with random variation of the fracture energy *w*_loc_(*x*, *y*), where the contact line is no longer perfectly circular (see Fig. 3A). The energy release rate *G* at a given point now depends on the whole shape of the contact *a*(*s*), where *s* is the length of the corresponding path along the contact circle. On the basis of the crack perturbation theory by Gao and Rice (*9*, *10*, *22*), we recently derived the approximate expression (*9*, *11*)

$$G\left(s\right)=G_{\text{JKR}}\left[a\left(s\right)\right]+c{\left(-\Delta_{s}\right)}^{1/2}a\left(s\right)$$ (3)

for the energy-release rate. Equation 3 has a simple interpretation: The adhesive contact line, *a*(*s*), behaves like an elastic line. The fractional Laplacian (−Δ*_s_*)^1/2^ of the contact shape *a*(*s*) (see also equation S35) yields a nonzero restoring force when the contact perimeter is no longer circular. This fractional Laplacian can be interpreted as a generalized curvature, and the prefactor *c* as the bending stiffness of the line. In the limit of a stiff line, *c* → ∞, the contact remains circular while in the opposite limit, *c* → 0, each point *s* along the contact perimeter can move independently because the restoring force disappears.

Section S1D derives Eq. 3 and shows that near equilibrium, where *G*(*s*) = *w*_int_, the bending stiffness *c* of the elastic contact line is equal to *w*_int_. Note that counterintuitively, the bending stiffness does not depend on the elastic modulus of the bulk but only on the intrinsic work of adhesion. Equations 2 and 3 describe the perimeter of the contact as an elastic line pinned by the random field *w*_loc_(*x*, *y*) and thereby establish an analogy between adhesion and other depinning phenomena (*12*–*14*, *23*).

The numerical solution of Eqs. 2 and 3 (see section S2) on a random field *w*_loc_(*x*, *y*) with a lateral correlation of length *d* yields force-area curves similar to those of our axisymmetric model (Fig. 3, B and C). The key difference is that the contact line now advances and recedes in jumps (Fig. 3A) that are localized over a characteristic length ℓ, the Larkin length (*12*–*14*, *23*, *24*). Between these jumps, the contact line is pinned. At the same rigid-body penetration, pinning occurs at lower contact radii during approach than during retraction, leading to a hysteresis in apparent adhesion described by two JKR curves with constant apparent work of adhesion *w*_appr_ and *w*_retr_ (Fig. 3C), similar to the curves obtained from our one-dimensional (1D) axisymmetric model (Fig. 2D).

Our numerical data in fig. S5 show that the magnitude of hysteresis, $w_{\text{retr}}-w_{\text{appr}}\propto w_{\text{rms}}^{2}$ , where $w_{\text{rms}}^{2}=\left\langle {\left(w_{\text{loc}}-\left\langle w_{\text{loc}}\right\rangle \right)}^{2}\right\rangle$ , is the variance of the random field *w*_loc_. To understand this expression, we first discuss the virtual limit *c* → 0 where the line is floppy and deviations from circularity are not penalized. Floppy lines (*c* < *w*_rms_) can freely distort and meander along valleys during approach (green line in Fig. 3D) and peaks during retraction (purple line). Because of this biased sampling of the work of adhesion along the line, the contact radius is larger during retraction than during approach. In this individual-pinning limit (*14*, *25*, *26*), each angle θ along the contact perimeter independently yields our 1D model and we obtain *w*_retr_ − *w*_appr_ ∝ *w*_rms_. In the opposite limit, *c* → ∞, the line is stiff and the contact remains circular (dashed line), randomly sampling as many regions of low and high adhesion. The fluctuations average out along the perimeter so that there is no hysteresis, *w*_retr_ − *w*_appr_ = 0. The contact radius is then obtained from the JKR expression evaluated for the spatially averaged work of adhesion, 〈*w*_loc_〉.

Our simulations (and experiments as shown below) are in an intermediate regime characterized by local jumps over length ℓ or *N* = ℓ/*d* pinning sites. The line is effectively rigid over the Larkin length ℓ and hence samples a coarse-grained work-of-adhesion field *w*^(ℓ)^ with $w_{\text{rms}}^{\left(\ell \right)}=w_{\text{rms}}/\sqrt{N}$ because the fluctuations average out stochastically over the rigid sections. From the line elasticity, Eq. 3 and section S1D, we obtain that an excursion of the contact line by distance δ*a* over this length leads to a restoring force δ*G* ∝ *c*δ*a*/ℓ, which must balance $w_{\text{rms}}^{\left(\ell \right)}$ . We note that δ*a* ≈ *d*, which is the distance to the closest local stable configuration (*12*, *13*). The equilibrium condition $\delta G=w_{\text{rms}}^{\left(\ell \right)}$ then yields

$$N\propto {\left(c/w_{\text{rms}}\right)}^{2}$$ (4)

where we used ℓ = *Nd*. This means that the magnitude of the hysteresis must scale as

$$w_{\text{retr}}-w_{\text{appr}}\propto w_{\text{rms}}^{\left(\ell \right)}\propto w_{\text{rms}}^{2}/c$$ (5)

exactly as observed in our simulations. Identical results were obtained previously for cracks in heterogeneous media (*14*, *27*).

### Topographic roughness

**Fig. 4.**
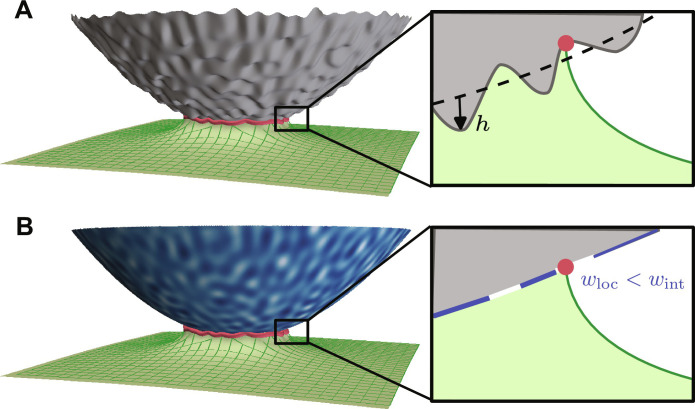
Mapping topographic roughness to equivalent chemical heterogeneity. The contact of a rough sphere (**A**) is equivalent to the contact of a sphere with a work-of-adhesion heterogeneity *w*_loc_ (**B**). The solid is stretched at the crack tip and surface roughness perturbs this elastic deformation. The associated perturbation of the elastic energy can equivalently be described by fluctuations of the work of adhesion.

The final step in describing the adhesion hysteresis on real surfaces is to relate the random height variations *h*(*x*, *y*), which describe the rough topography, to spatial variations in the fracture energy *w*_loc_(*x*, *y*). For this, we need to consider excursions of the contact line normal to the surface in addition to the lateral excursions that are described by the contact radius *a*(θ) (see Fig. 4). First, note that the solid is always dilated near the crack tip. To conform to a valley, the elastic solid needs to stretch even more, requiring elastic energy. Using the same arguments that lead to Eq. 1, this additional elastic energy manifests as an effectively decreased local work of adhesion *w*_loc_. Conversely, conforming to a peak decreases the overall strain near the crack tip and releases elastic energy, leading to an increased equivalent work of adhesion. While this intuitive picture approximately describes the relationship between heights and local adhesion, the quantitative value of the local adhesion *w*_loc_ depends nonlocally on the topographic field *h*(*x*, *y*) via an integral transformation derived in section S1 (B and C). Section S3 also shows that a crack-front simulation that uses *w*_loc_(*x*, *y*) yields results virtually indistinguishable from an exact boundary-element calculation on the rough topography *h*(*x*, *y*).

### Comparison to experiments

**Fig. 5.**
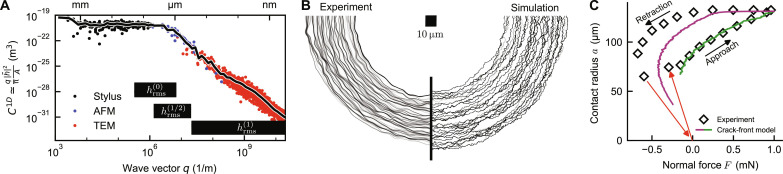
Crack-front pinning explains adhesion hysteresis on real-world surfaces with multiscale roughness. (**A**) Power spectral density (PSD) of a nanocrystalline diamond (NCD) film extracted from more than 60 measurements (*16*), combining stylus profilometry, atomic force microscopy (AFM), and transmission electron microscopy (TEM). Black bars indicate the range of scales that dominate $h_{\text{rms}}^{\left(\alpha \right)}$ (Eq. 8). (**B**) Position of the perimeter in the contact between a rubber sphere and a rough surface during approach. The perimeters on the left side are extracted from the experiment on NCD shown in Fig. 1, and the right side shows equilibrium positions of the perimeter in a crack perturbation simulation (see sections S1 and S2) on random roughness similar to NCD. The contact perimeter is pinned where the black lines are close to each other, while regions with a low density of lines indicate where the contact perimeter accelerates during an instability. The simulation predicts instabilities of various sizes, reaching a lateral extent up to several tens of micrometers. In the experiment, only the largest instabilities and the largest features of the contact line are visible because of the limited resolution of the camera and because we removed image noise using a spatially averaging filter. The positions of the perimeter are shown from jump into contact until the force reaches 0.64 mN. (**C**) Contact radius and normal force during approach and retraction of the experiment (diamonds) and simulation (continuous line) shown in (B). We extracted the intrinsic work of adhesion *w*_int_ = 63 mJ/m^2^ used in the simulation by fitting the work of approach. Figure S6 shows that the PSD of the synthetic random roughness used in the simulation is close to the PSD of NCD at the length scales that dominate $h_{\text{rms}}^{\left(1/2\right)}$.

We contacted a rough nanodiamond film with a polydimethylsiloxane (PDMS) hemispherical lens while optically tracing the contact perimeter (see Materials and Methods). The nanodiamond film was characterized from atomic to macroscopic length scales using a variety of techniques, as described in (*15*, *16*). The resulting power spectral density (PSD) (*28*) comprehensively describes the topography of the film and is shown in Fig. 5A. This experiment is compared to a simulation carried out on a roughness field with an identical PSD, leaving *w*_int_ as the only free parameter. We determine *w*_int_ by fitting the approach curves of the simulation and of the experiment. This yields *w*_int_ = 63 mJ m^−2^, within the range expected for van der Waals interaction.

Our experiments show the same instabilities as the simulations. The trace of the contact line in Fig. 5B shows the jerky motion of the line for both, with comparable amplitudes of deviations from the ideal contact circle. Videos of the contact area in the indentation experiment (movie S1) show the stick-slip motion of the contact line, similar to our simulations and to observations by Lyashenko and Pohrt (*29*) on contact with rubber membranes. The fundamental hysteresis mechanism in our model, elastic instabilities, and stick-slip motion of the contact line are clearly present in the experiment.

Measurements of the mean contact radius as a function of normal force also agree with our simulation results (Fig. 5C). While the simulation was adjusted to follow the experimental data during the approach, the match is almost perfect by adjusting only a single parameter, *w*_int_. The functional form of the experiment during approach is hence JKR-like with an apparent *w*_appr_ ≃ 29 mJ/m^2^. During retraction, we observe the same phenomenology: From the point of largest normal force, the sphere retracts first at a constant contact radius before starting to follow a JKR-like curve with an increased work of adhesion *w*_retr_ ≃ 106 mJ/m^2^. While the simulation retracts at slightly different forces, corresponding to ≃71 mJ/m^2^, the order of magnitude of the hysteresis is correctly predicted from our simple elastic model.

## DISCUSSION

The matching order of magnitude between our model and the experiment shows that elastic instabilities are an important contribution to the adhesion hysteresis of the real contact. The larger hysteresis in the experiment may originate from other dissipation mechanisms. When repeating the experiment in the absence of surface roughness on hydrophobically functionalized surfaces (see Materials and Methods and fig. S7), a hysteresis of *w*_appr_ − *w*_retr_ ≈ 21 mJ/m^2^ remains. This value corresponds to half of the difference between the measured and the predicted hysteresis. Because these experiments are carried out on smooth contacts, this remaining hysteresis must come from material-specific dissipation processes, most likely viscoelasticity.

We expect the viscoelastic contribution to the hysteresis in the rough contact to be at most as large as on the smooth surface. Viscoelastic energy dissipation increases the apparent work of adhesion at high crack speeds (*30*, *31*). While the average crack velocities are similar in both experiments, in the rough contact, the local velocity deviates substantially from the average. It either vanishes when the crack front is pinned or is orders of magnitude higher than the average during an instability. When it is pinned, the crack front is immobile and the viscoelastic effects are lower than in the smooth reference experiment. During an instability, the crack accelerates, until dissipation mechanisms such as viscoelasticity become active. However, the total energy dissipated during the instability is predetermined by the energy difference between the quasi-static pinned configurations just before and just after the instability; see Fig. 3A. Any viscoelastic contribution to the adhesive force is determined by the pinned configurations of the crack, where viscoelastic effects are at most as large as in the smooth contact.

Besides material-specific dissipation, quantitative differences could come from approximations or intrinsic assumptions of our model, such as the assumption of fully conforming contact. Contacts conform if the energy needed to fully conform to the surface roughness is much lower than the gain in surface energy, *e*_el_ ≪ *w*_int_ (*3*, *32*). For *e*_el_ ≲ *w*_int_ such as in our experiments, deep valleys may not enter into contact and leave penny-shaped microcracks within the perimeter of the contact. These microcracks likely increase the adhesion hysteresis and the pull-off force because the movement of the additional contact lines in the interior of these microcracks will also be subject to pinning by the topographic roughness. However, many experiments report a decrease of pull-off force with increasing roughness as, for example, reported in the classic adhesion experiment by Fuller and Tabor (*33*). These experiments may be within this limit *e*_el_ > *w*_int_, where only partial contact is established within the contact circle (*4*, *32*, *34*–*36*). Unlike the theory presented here for conforming contacts and our understanding of nonadhesive contact (*3*), there is presently no unifying theory that quantitatively describes adhesion in partial contact. Large-scale simulations with boundary-element methods are needed to better understand this intermediate regime (*4*, *35*–*39*).

We now show that simple analytic estimates can be obtained from our crack-front model. The equivalent work-of-adhesion field has the property that its mean corresponds to the Persson-Tosatti expression (Eq. 1). Furthermore, it has local fluctuations with amplitude $w_{\text{rms}}=\sqrt{2w_{\text{int}}e_{\text{el}}}$ which determine the adhesion hysteresis; see Eq. 5. This equation means that the main parameter determining the hysteresis is *e*_el_. We carried out crack-front simulations on self-affine randomly rough topographies (Fig. 3C and section S4) to confirm that the apparent work of adhesion during approach and retraction is given by

$$w_{\overset{\text{retr}}{\text{appr}}}=w_{\text{int}}-e_{\text{el}}\pm ke_{\text{el}}$$ (6)

and to determine the numerical constant *k* ≃ 3. We parametrically varied the roughness to confirm that the main parameter determining adhesion hysteresis is *e*_el_.

This expression allows us to connect the adhesion hysteresis to the statistical parameters of the rough topography. The elastic energy for fully conformal contact can be written as

$$e_{\text{el}}=\frac{E\prime }{4}{\left[h_{\text{rms}}^{\left(1/2\right)}\right]}^{2}$$ (7)

where *E*′ is the elastic contact modulus (*40*) and $h_{\text{rms}}^{\left(1/2\right)}$ is a geometric descriptor of the rough topography. In terms of the 2D PSD (*28*) *C*^iso^, we define

$${\left[h_{\text{rms}}^{\left(\alpha \right)}\right]}^{2}=\frac{1}{4\pi^{2}}\int d^{2}q\;{\left|\vec{q}\right|}^{2\alpha }C^{\text{iso}}(|\vec{q}|)$$ (8)

where $\vec{q}$ is the wave vector. This expression contains the root mean square (rms) amplitude of the topography, $h_{\text{rms}}^{\left(0\right)}$ , the rms gradient of the topography, $h_{\text{rms}}^{\left(1\right)}$ , as well as arbitrary derivatives of order α. The elastic energy is given by the roughness parameter $h_{\text{rms}}^{\left(1/2\right)}$ , which is intermediate between rms heights and rms gradients.

For most natural and engineered surfaces, $h_{\text{rms}}^{\left(1/2\right)}$ depends on the large scales, like the rms height, because of their Hurst exponent *H* > 0.5 (*5*, *41*–*43*). Our model is then consistent with the increase in pull-off force with *h*_rms_ reported in (*19*, *44*). We note that most measurements report insufficient details on surface roughness to allow definite conclusions on the applicability of a certain contact model. The range of length scales that dominate $h_{\text{rms}}^{\left(1/2\right)}$ in our own experiments is at the transition between power-law scaling and the flat roll-off at 2 μm, a length scale that is accessible with an atomic-force microscope. We illustrate the respective scales that contribute to $h_{\text{rms}}^{\left(\alpha \right)}$ in Fig. 5A.

The work performed on a soft indenter during the approach-retraction cycle is dissipated in elastic instabilities triggered by surface roughness. The dissipated energy is the difference in energy between the pinned configurations just before and just after the instability. This pinning of the contact line explains why adhesion is always stronger when breaking a soft contact than when making it, even in the absence of material-specific dissipation. Roughness peaks increase local adhesion, which pins the contact line and increases the pull-off force. By describing rough adhesion as the pinning of an elastic line, we were able to derive parameter-free, quantitative expressions for the hysteresis in terms of a simple statistical roughness parameter. This analysis paves the way to better understanding the role of surface roughness in adhesion and provides guidance for which scales of roughness to control to tune adhesion.

## MATERIALS AND METHODS

### Crack perturbation simulations

We use a crack perturbation model (*9*–*11*) to compute the energy release rate at the perimeter of the contact and solve for equilibrium with the local (equivalent) fracture energy *w*_loc_ using the algorithm by Rosso and Krauth (*45*). The derivation of the crack perturbation equations and the mapping from surface roughness to the equivalent work-of-adhesion heterogeneity is provided in the Supplementary Materials.

### Rough substrate

We contacted the PDMS lens against a nanocrystalline diamond (NCD) film of known roughness. The diamond film was deposited on a silicon wafer by chemical vapor deposition and, subsequently, hydrogen-terminated to avoid polar interactions and hydrogen bond formation between the PDMS lens and the rough substrate. The roughness of the film was determined by combining measurements from the millimeter to atomic scales using a stylus profilometer, atomic force microscopy (AFM), and transmission electron microscopy (TEM). The full experimental dataset along with the averaged PSD shown in Fig. 5B is available online (*46*). Details on the film growth and the multiscale topography characterization are provided in (*15*, *16*).

Evaluating Eq. 8 requires the 2D or isotropic PSD of the surface topography, while only the 1D PSD is known. Following (*7*, *28*), we converted the 1D PSD *C*^1D^ to the isotropic 2D PSD using the approximation *C*^iso^(*q*) ≃ π*C*^1D^(*q*)/*q*.

### Synthesis of PDMS hemispheres

We synthesized PDMS hemispheres of 0.7-MPa Young’s modulus by hydrosilylation addition reaction. Vinyl-terminated PDMS V-41 (weight-averaged molar mass *M_w_* = 62,700 g/mol) as a monomer, tetrakis(dimethylsiloxy)silane as a tetra-functional cross-linker, and platinum carbonyl cyclo-vinyl methyl siloxane as a catalyst were procured from Gelest Inc. Monomer and cross-linker were first mixed in a molar ratio of 4:4 in an aluminum pan. The catalyst was added as 0.1 wt % of the total mixture, and lastly, the batch was degassed in a vacuum chamber for 5 min. Hemispherical lenses were cast on fluorinated glass dishes using a needle and a syringe and cured at 60°C for 3 days. Since the PDMS mixture has a higher surface energy than the fluorinated surface, the drops maintain a contact angle on the surface, giving the shape of a hemispherical lens. After the curing reaction, the lenses were transferred to cellulose extraction thimble for Soxhlet extraction where toluene refluxes at 130°C for 48 hours. PDMS lenses were again transferred to a fluorinated dish and dried in air for 12 hours. Last, the lenses were vacuum-dried at 60°C for 16 hours and then used for experiments. Before using the lens in the experiment, the radius of curvature *R* = 1.25 mm was measured by fitting a three-point circle to a profile image obtained using an optical microscope (Olympus).

### Indentation experiment

We measured the force and area during the approach and retraction of a PDMS hemisphere against a rough diamond film using the setup of Dalvi *et al.* (*7*). The lens and the substrate were approached at a constant rate of 60 nm/s until a repulsive force of 1 mN, and then retracted with the same rate. The PDMS hemisphere is transparent, allowing simultaneous measurement of the force and of the contact area (Fig. 1B). The video recording, provided in movie S1, has a frame interval of 0.3 s, but Fig. 5C shows values for the force and contact radius at intervals of ≈30 s. To remove the influence of roughness, we also carried out reference experiments on a flat silicon wafer covered with hydrophobic octadecyltrichlorosilane (see fig. S7). The Young’s modulus *E* = 0.7 MPa of the PDMS sphere was obtained by fitting the JKR theory to these experiments [see also (*7*)].

### Extraction of contact line from video

We extracted the perimeter from each time frame of the video of the contact area. The contact area appears as a bright region in the video, and we defined the contact perimeter as a contour line of a fixed level of gray. At the length scale of a few pixels, the position of the line is affected by noise in the image. To reduce the effect of noise on the position of the line, we subtracted the image of the contact area at maximum penetration and subsequently applied a spatial Gaussian filter of variance 2 pixels. The lines shown in Fig. 5B therefore only reflect the position of the perimeter on coarse scales. Movie S2 shows that these lines match the shape of the contact area at large scales and follow the same intermittent motion. The original video is available in the Supplementary Materials (movie S1).

## Supplementary Materials

### This PDF file includes:

Sections S1 to S4 Figs. S1 to S7 Legends for movies S1 and S2 References
