# Supplementary material for: Why soft contacts are stickier when breaking than when making them
Source: Sci Adv. 2024 Mar 6;10(10):eadl1277. doi: 10.1126/sciadv.adl1277 (PMC10917342; doi:10.1126/sciadv.adl1277)
Supplement: Supplementary file 1 — Sections S1 to S4 Figs. S1 to S7 Legends for movies S1 and S2 References [file sciadv.adl1277_sm.pdf]

Science Advances
AAAS

Supplementary Materials for

Why soft contacts are stickier when breaking than when making them

Antoine Sanner *et al.*

Corresponding author: Lars Pastewka, lars.pastewka@imtek.uni-freiburg.de

Sci. Adv. **10**, eadl1277 (2024)
DOI: 10.1126/sciadv.adl1277

The PDF file includes:

Sections S1 to S4
Figs. S1 to S7
Legends for movies S1 and S2
References

Other Supplementary Material for this manuscript includes the following:

Movies S1 and S2

SUPPLEMENTARY TEXT

S1. CRACK-FRONT MODEL

Our goal is to model the contact of a rough sphere on a deformable elastic flat (Fig. S1a). The contact perimeter of the adhesive contact between a smooth sphere and flat can be regarded as a circular crack (Fig. S1b). This is the basis of the Johnson, Kendall and Roberts (JKR) model for adhesion (2). JKR derived an expression for the elastic energy release rate G_{JKR} for this spherical geometry, and balanced it with the intrinsic work of adhesion, $G_{\mathrm{JKR}} = w_{\mathrm{int}}$. Here, we extend this result to rough spheres, where the crack shape deviates from circularity. Surface roughness perturbs the shape of the crack in the surface normal direction (Figs. 4 and S1c). This perturbs the local balance of energy, leading to additional deviation of the crack shape in direction parallel to the surface (Fig. S1d).

Figure S1 illustrates this decomposition in terms of the energy release rate G. The surface roughness h locally perturbs the elastic energy by $G_{\perp}$ (Fig. S1c) (49) and the perimeter distorts within the plane to satisfy equilibrium with the uniform work of adhesion w_{int} (Fig. S1d). As we show in detail in this supplementary material, we describe the effect of surface roughness by an equivalent work-of-adhesion field

$$w_{\mathrm{loc}}([h]; a(\theta), \theta) = w_{\mathrm{int}} - e_{\mathrm{el}}([h]) - G_{\perp}([h]; a(\theta), \theta), \tag{S1}$$

where e_{el} is the elastic energy required to fully conform to the surface roughness and the square brackets indicate a functional dependency.

The effect of the in-plane deflection on the elastic energy $G_{\parallel}$ was derived by Gao and Rice (9) and later extended by us to spheres (11). In our simulations, the equilibrium condition

$$w_{\mathrm{loc}}([h]; a(\theta), \theta) = G_{\mathrm{JKR}}(b, a(\theta)) + G_{\parallel}([a], \theta) \tag{S2}$$

determines the contact radius with $\mathcal{O}(h^2)$ errors in the strength of the disorder. The left-hand side represents the driving force to increase the contact radius that fluctuates according to the surface roughness, while the right-hand side represents the elastic response of the line that only depends on the spherical geometry and the material properties. The numerical implementation follows Refs. (11, 45) and is summarized in supplementary material S2. We

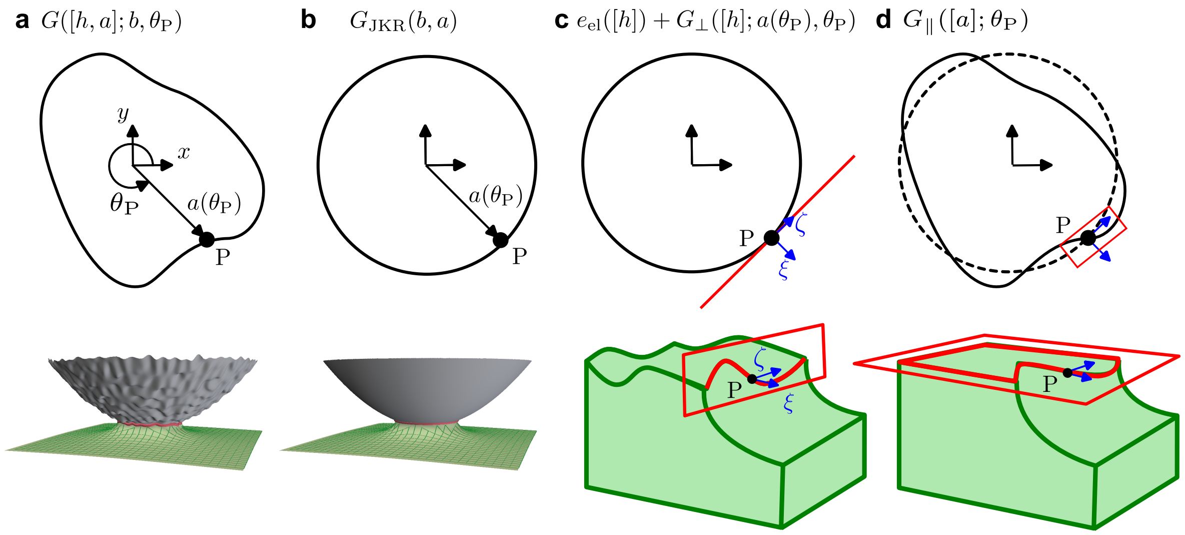

FIG. S1. **Contact of a sphere of radius R with superposed roughness $h(x, y)$. (a)** Because of surface roughness, the contact perimeter is no longer circular. We describe it by the contact radius $a(\theta)$, the planar distance between the tip of the sphere and the perimeter of the contact. The energy release rate G at the point P along the crack is decomposed into three contributions, $G = G_\mathrm{JKR} + (e_\mathrm{el} + G_\perp) + G_\parallel$, illustrated in panels (b) to (d). **(b)** The energy release rate $G_\mathrm{JKR}(b, a)$ for the smooth contact is given by the theory of Johnson, Kendall and Roberts (2). **(c)** Surface roughness leads to out-of-plane displacements of the contact perimeter. This increases the average energy release rate by e_el and leads to additional local fluctuations $G_\perp([h]; a(\theta_\mathrm{P}), \theta)$. Here, e_el is the elastic energy needed to fully conform to surface roughness. **(d)** The in-plane deflection of the perimeter from circularity leads to the additional contribution $G_\parallel([a], \theta)$.

validate our equations by comparing crack-front simulations to boundary element method simulations in supplementary material S3. Equations (S1) and (S2) establish the equivalence between the adhesion of rough spheres and the classic problem of the pinning of an elastic-line by quenched disorder (12–14, 24, 27, 45).

A. Axisymmetric contact: The JKR model

We consider the contact of a sphere (to be exact, a paraboloid) adhering an elastic half-space at a fixed rigid-body penetration b (Fig. S1a). This case can be mapped to the contact of two spheres with the same composite radius R and contact modulus E' (40). When only one half-space deforms, $E' = E/(1 - \nu^2)$, where E is Young's modulus and ν is Poisson's ratio. Fracture mechanics typically considers the contact of two elastic half-spaces where $E' = E/2(1 - \nu^2)$. We assume the contact is frictionless and consider only vertical

displacements of the half space.

The equilibrium radius and force for a perfect sphere against the axisymmetric work-of-adhesion heterogeneity $w_{\text{loc}}(a)$ is given by the JKR theory (2, 47, 48). JKR described the adhesion of a paraboloid with radius R by superposing the displacement and the stress fields of the nonadhesive Hertzian contact (50) and the circular flat punch under tensile load (51).

The contact pressures p have a tensile singularity as the distance to the edge of the contact $-\xi$ goes to 0,

$$p(\xi) = -K_{\text{JKR}}/\sqrt{2\pi(-\xi)} + \mathcal{O}((-\xi)^{1/2}), \tag{S3}$$

with the stress intensity factor

$$K_{\text{JKR}} = \left(\frac{a^2}{R} - b\right) \frac{E'}{\sqrt{\pi a}}. \tag{S4}$$

Here and below we use the subscript JKR to indicate the circular contact to a smooth sphere. The energy release rate depends solely on the amplitude of this singularity (52)

$$G_{\text{JKR}} = K_{\text{JKR}}^2/(2E') \tag{S5}$$

and the equilibrium condition $G_{\text{JKR}}(b, a) = w_{\text{loc}}(a)$ yields the contact radius. The normal force is given by

$$F_{\text{JKR}}(a, b) = \frac{4E'}{3R}a^3 + 2aE'\left(b - \frac{a^2}{R}\right). \tag{S6}$$

At equilibrium, where $G_{\text{JKR}} = w_{\text{loc}}$, the relationship between force and contact area is given by

$$F_{\text{JKR}}(a) = \frac{4E'}{3R}a^3 - \sqrt{8\pi w_{\text{loc}}(a)E'a^3}. \tag{S7}$$

Once nondimensionalized using distinct vertical and lateral length units, the JKR contact is parameter free (48, 53, 54), and we present our numerical results in the nondimensional units defined in Refs. (47, 48). Specifically, lengths along the surface of the half-space (e.g., the contact radius) are normalized by $(3\pi w_{\text{int}} R^2/4E')^{1/3}$, lengths in vertical direction (e.g., displacements) by $(9\pi^2 w_{\text{int}}^2 R/16E'^2)^{1/3}$ and normal forces by $\pi w_{\text{int}} R$. The equations are in dimensional form but can be nondimensionalized by substituting $R = 1$, $w_{\text{int}} = 1/\pi$ and

$E' = 3/4$.

B. Circular contact with surface roughness: Out-of-plane perturbation of the elastic energy release rate

We now determine the energy release rate at the perimeter of the contact with a *rough* sphere but where the contact perimeter remains circular (Fig.S1c). We denote the respective energy release by $G_\circ([h]; b, a, \theta)$, where the brackets indicate a functional dependency on the height field $h(x, y)$ that describes the roughness. Out-of-plane deflections of the surface of the solid make the elastic energy release rate $G_\circ([h]; b, a, \theta)$ fluctuate along the contact perimeter, and θ parameterizes the angle along the perimeter of the circular crack front. In the main text and in our simulations, we formally describe this perturbation of the energy release rate by the equivalent work of adhesion w_{loc}. In order to justify this mapping, we first discuss the *true* elastic energy release rate $G_\circ$ and show that the effects of the spherical geometry, surface roughness and in-plane distortion of the crack front are decoupled.

The JKR contact is the superposition of the (adhesive) flat punch (51) and the Hertz solution (50). For the rough sphere, we now additionally superpose the stresses and displacements needed to conform to the surface roughness. We do not need to determine the whole distribution of contact stresses because the energy release rate only depends on the stress intensity factor at the contact edge via Irwin's relation (52)

$$G_\circ([h]; b, a, \theta) = \left\{K_{\mathrm{JKR}}(b, a) + K_\perp([h]; a, \theta)\right\}^2 / (2E'), \tag{S8}$$

where $K_\perp$ captures the effect of roughness. $K_\perp$ can be thought of as the stress intensity factor in the conforming contact of a circular flat punch with roughness h at zero external load. Note that the stress intensity factors of the JKR solution and the influence of roughness can be superposed linearly, because in linear elasticity we can simply superpose stresses originating from different geometric contributions.

1. Stress intensity factor caused by roughness at the tip of a semi-infinite crack

We compute $K_\perp$ approximately by treating the contact as a semi-infinite crack (Fig. S2a,b), i.e. the roughness features are small compared to the contact radius. We describe the semi-infinite crack in the coordinate system ξ, ζ, where ξ points in the normal to the crack front with $\xi < 0$ in the contacting area. ζ points parallel to it. This is essentially a locally rotated coordinated system at the angle θ on the crack, as shown in Fig. S1c. The semi-infinite crack hence represents a small subsection of the circular perimeter centered at $\xi = 0$ and $\zeta = 0$.

We compute the stress intensity factor by a classic superposition (55, 2.6.4 Full Stress Field for Mode-I Crack in an Infinite Plate), where we first compute the pressures needed to conform the surface roughness in the absence of a crack (Fig. S2c) and subsequently cancel out these pressures on the crack faces ($\xi > 0$) (Fig. S2d). Loading the crack faces while keeping the displacements fixed in the contact area ($\xi < 0$) leads to the stress intensity factor $\bar{K}_\perp(\zeta)$. The bar over $\bar{K}_\perp$ indicates that the result is valid for a straight crack.

The pressures needed to conform to the surface roughness in the infinite contact are (5, 56),

$$\tilde{p}_\infty(\vec{q}) = \frac{E'}{2}|\vec{q}|\tilde{h}(\vec{q}), \tag{S9}$$

where $\vec{q} = (q_x, q_y)$ is the wavevector and the tilde denotes the Fourier transform,

$$\tilde{h}(q_x, q_y) = \int_{-\infty}^{\infty} \mathrm{d}x\,\mathrm{d}y\, e^{-i(q_x x + q_y y)} h(x, y). \tag{S10}$$

The stress intensity factor at the edge of the contact results from the crack-face loading needed to cancel p_∞ outside the contact area,

$$\bar{K}_\perp([h]; x, y) = \int_x^\infty \mathrm{d}x_\mathrm{P} \int_{-\infty}^\infty \mathrm{d}y_\mathrm{P}\, k(x_\mathrm{P} - x, y_\mathrm{P} - y)\left\{-p_\infty([h]; x_\mathrm{P}, y_\mathrm{P})\right\}. \tag{S11}$$

The quantity $\bar{K}_\perp$ is the stress intensity factor at position y along the tip of a crack advanced to position x (Fig. S2a). The crack-face weight function (57)

$$k(\xi, \zeta) = \frac{\sqrt{2/\pi^3}\sqrt{\xi}}{\xi^2 + \zeta^2}, \tag{S12}$$

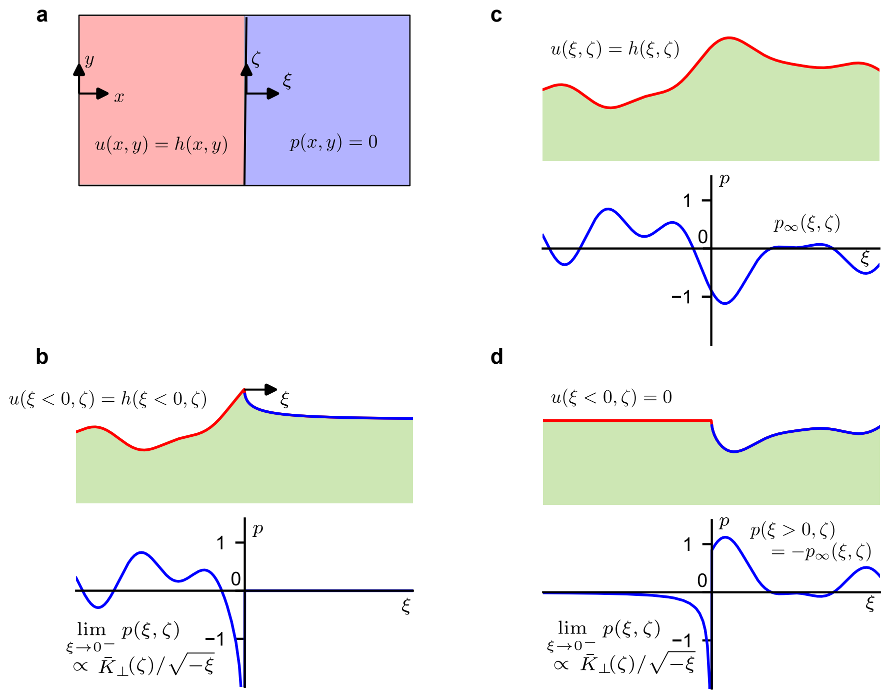

FIG. S2. **Stress intensity factor caused by surface roughness for a straight crack $\bar{K}_\perp$ using a superposition. (a)** We consider a semi-infinite crack located at x, for which the positive x and ξ directions point towards the cracked area. The local coordinate system ξ, ζ is centered on the crack tip, so that $\xi < 0$ corresponds to the contact area. **(b)** For $\xi > 0$, the surface is free to move vertically and the pressure $p = 0$. For $\xi < 0$, the solid fully conforms to the surface roughness so that the displacements u are prescribed to be equal to the heights h. Note that the positive direction for displacements and heights, corresponding to roughness peaks, points into the elastic halfspace (downwards). In the contact area, surface roughness causes fluctuating contact pressures $p(x, y)$ with stress intensity factor $\bar{K}_\perp(x, y)$. We compute $\bar{K}_\perp(x, y)$ by superposing the solutions of two elastic problems (c) and (d). **(c)** Displacements and pressures in an uncracked contact with the roughness h. The displacements $u(x, y) = h(x, y)$ cause the pressure distribution $p_\infty(x, y)$ **(d)** Semi-infinite crack with pressures applied on his crack faces. We apply the pressures $-p_\infty$ so that the pressures cancel out on the crack faces when superposing to (c). The displacements are 0 in the contact area so that the contact condition $u = h$ remains satisfied for $\xi < 0$ after superposition. Loading the crack faces at fixed displacements in the contact area causes the stress intensity factor. This stress intensity factor corresponds to $\bar{K}_\perp$ because there is no stress singularity in solution (c).

is the stress intensity factor at the origin of a semi-infinite crack caused by a unit point force at (ξ, ζ). Evaluating the convolution Eq. (S11) for each position of the crack x yields a two-dimensional field of stress intensity factors, which can be most easily represented in

terms of its Fourier modes,

$$\tilde{\bar{K}}_\perp(q_x, q_y) = -\frac{E'}{\sqrt{2}}\sqrt{iq_x + |q_y|}\tilde{h}(q_x, q_y) \tag{S13}$$

with

$$\bar{K}_\perp([h]; x, y) = \frac{1}{4\pi^2}\int\limits_{-\infty}^{\infty} \mathrm{d}q_x\,\mathrm{d}q_y\,\tilde{\bar{K}}_\perp(q_x, q_y)e^{i(q_x x + q_y y)}. \tag{S14}$$

Note that $\bar{K}_\perp$ has zero average (because of symmetry of the elastic surface response) and that the solids overlap where the stress intensity factor is negative. Our final result has no overlap provided that $|\bar{K}_\perp| < K_{\mathrm{JKR}}$. Anderson and Rice (49) derived an equation equivalent to Eq. (S13) to understand the interaction of crack tips with dislocations.

We now detail the steps leading from Eq. (S11) to Eq. (S13). The pressures needed to conform to the surface roughness in the infinite contact p_∞ are easier to express in Fourier space, see Eq. (S9). Using the Heaviside step function $\Theta(\xi)$, we now define the weight function on the whole plane as

$$f(\xi, \zeta) = \Theta(\xi)k(\xi, \zeta). \tag{S15}$$

This allows us to extend the integration bound on Eq. (S11) to infinity. The convolution theorem then yields the simple expression

$$\tilde{\bar{K}}_\perp(q_x, q_y) = \tilde{f}^*(q_x, q_y)(-\tilde{p}_\infty(q_x, q_y)), \tag{S16}$$

where the star is the complex conjugate. We now compute the Fourier transform of this generalized weight function

$$\tilde{f}(q_x, q_y) = \int\limits_{-\infty}^{\infty} \mathrm{d}\xi\,\mathrm{d}\zeta\,\Theta(\xi)k(\xi, \zeta)e^{-iq_x\xi}e^{-iq_y\zeta}. \tag{S17}$$

Using that

$$\int\limits_{-\infty}^{\infty} \mathrm{d}y\,e^{-iq_y y}k(x, y) = \int\limits_{-\infty}^{\infty} \mathrm{d}y\,e^{-iq_y y}\frac{\sqrt{2/\pi^3}\sqrt{x}}{x^2 + y^2} = \sqrt{\frac{2}{\pi}}\frac{e^{-x|q_y|}}{\sqrt{x}} \tag{S18}$$

and evaluating the step function, we get a classic Laplace transform (58, Eq. 29.3.4)

$$\tilde{f}^*(q_x, q_y) = \sqrt{2} \int_0^\infty \mathrm{d}x \, \frac{1}{\sqrt{\pi x}} e^{-(|q_y| - i q_x)x} = \frac{\sqrt{2}}{\sqrt{|q_y| - i q_x}}. \tag{S19}$$

Inserting Eq. (S9) into Eq. (S16) and using that $|q| = \sqrt{|q_y| - iq_x}\sqrt{|q_y| + iq_x}$ yields Eq. (S13).

C. Circular contact with surface roughness: Equivalence between topographic roughness and chemical heterogeneity

We now switch from the straight crack back to the contact of a sphere. The first step is to approximate the stress intensity factor $K_\perp$ by the result for the straight crack, $\bar{K}_\perp$, obtained above. This approximation requires us to rotate the straight crack to be tangential to the contact circle, i.e. to rotate it by the angle θ that gives the circumferential position (Fig. S1c). Note that for isotropic random fields, this rotation becomes inconsequential and we do not carry it out for the results shown in the main text. We do carry out this rotation when comparing the crack-front results to the boundary element method, shown in Sec. S3 below. Approximating $K_\perp$ by $\bar{K}_\perp$ is not strictly necessary, since integral expressions for the stress intensity factor for a circular flat punch with surface roughness exist (59–61). The advantage of the expression for the straight crack Eq. (S13) is that it links the statistics of the chemical heterogeneity to the statistics of the surface roughness in a simple way, while the expressions for the circular contact are more difficult to evaluate and to interpret.

We now rewrite Eq. (S8) as

$$G_\circ([h]; b, a, \theta) = G_{\mathrm{JKR}}(b, a) + K_{\mathrm{JKR}}(b, a) K_\perp([h]; a, \theta)/E' + K_\perp^2([h]; a, \theta)/(2E'). \tag{S20}$$

The term $K_\perp$ is stochastic, as it describes the influence of surface roughness, which is typically a random field. Since $K_\perp$ is linear in h, its spatial average $\langle K_\perp \rangle_{a,\theta}$ vanishes. For a random field with a short correlation length, even partial averages over just the angle θ must vanish. This means the middle summand in Eq. (S20) does not contribute to the average energy release rate. However, the variance $\langle K_\perp^2 \rangle_{a,\theta}$ must be positive and nonzero. Parseval's

theorem tells us that

$$\left\langle K_\perp^2/2E'\right\rangle_{a,\theta} = \frac{E'}{16\pi^2}\int \mathrm{d}q_x\,\mathrm{d}q_y\,|q|C^{\mathrm{2D}}(q_x,q_y) = e_{\mathrm{el}}, \tag{S21}$$

where $C^{\mathrm{2D}}(q_x,q_y) = (L_xL_y)^{-1}|\tilde{h}(q_x,q_y)|^2$ is the power-spectral density of the heights (28) and L_x, L_y are the period of the system in the respective direction. Note that while we consider the limit of an infinite system size $L_x, L_y \to \infty$, C^{2D} remains finite. The variance gives the elastic energy e_{el} for fully conformal contact. The average of Eq. (S20) then becomes

$$\langle G_\circ\rangle_{a,\theta} = G_{\mathrm{JKR}} + e_{\mathrm{el}}. \tag{S22}$$

This equation is equivalent to a classic result by Persson and Tosatti (5). They approximated equilibrium by $\langle G_\circ\rangle_{a,\theta} = w_{\mathrm{int}}$. Formally, this can be described by the equilibrium of a smooth sphere with the uniform equivalent work of adhesion $w_{\mathrm{loc}} = w_{\mathrm{int}} - e_{\mathrm{el}}$, where $G_\circ = G_{\mathrm{JKR}}$. This approximation only works in the adiabatic limit. Fluctuations become crucial when they are able to pin the crack front and trigger instabilities.

We now show how to generalize Persson and Tosatti's result to describe local fluctuations. This means we need to consider the effect of the second term in Eq. (S20),

$$G_\perp([h];b,a,\theta) = K_{\mathrm{JKR}}(b,a)K_\perp([h];a,\theta)/E', \tag{S23}$$

that disappears in the average but represents the leading-order effect of roughness on the fluctuations of $G_\circ$. $G_\perp$ depends on the geometry and position of the indenter via K_{JKR}. This coupling between macroscopic boundary conditions and the microscopic disorder is a second-order effect of the roughness, which we can neglect because our final equilibrium equation determines the crack shape with first-order accuracy only. To first order in h, we approximate $K_{\mathrm{JKR}} \approx \sqrt{2w_{\mathrm{int}}E'}$, yielding

$$G_\perp([h];a,\theta) \approx \sqrt{2w_{\mathrm{int}}/E'}K_\perp([h];a,\theta). \tag{S24}$$

This first-order approximation allows us to describe the effect of surface roughness by the

equivalent quenched disorder in work of adhesion

$$w_{\text{loc}}([h];a,\theta) = w_{\text{int}} - e_{\text{el}}([h]) - G_{\perp}([h];a,\theta). \tag{S25}$$

The equivalent work of adhesion Eq. (S25) contains only the essential leading-order contributions of the roughness and is independent of the macroscopic geometry, so that our results generalize to other adhesion setups where our approximations are valid.

Our mapping to an equivalent work of adhesion establishes a link to the pinning of elastic lines by quenched disorder. Theoretical work on the pinning of elastic lines (12–14, 27) allows us to link the hysteresis in apparent adhesion to the root-mean-square (rms) fluctuations of w_{loc}. Inserting Eq. (S13) into Eq. (S24) and (S25) yields

$$w_{\text{rms}} = \sqrt{\left\langle \left(w_{\text{loc}} - \langle w_{\text{loc}} \rangle_{a,\theta} \right)^2 \right\rangle_{a,\theta}} = 2\sqrt{e_{\text{el}} w_{\text{int}}} = h_{\text{rms}}^{(1/2)} \sqrt{E' w_{\text{int}}}. \tag{S26}$$

The quantity $h_{\text{rms}}^{(1/2)}$ is the rms half-derivative (or quarter-fractional Laplacian) given by

$$\left[h_{\text{rms}}^{(\alpha)} \right]^2 = \frac{1}{4\pi^2} \int \mathrm{d}^2 q \, |\vec{q}|^{2\alpha} C^{2\text{D}}(\vec{q}) \tag{S27}$$

with $\alpha = 1/2$.

The fluctuations of w_{loc} are linear in the roughness amplitudes. Since our assumption of fully conformal contact requires that $e_{\text{el}} < w_{\text{int}}$, w_{rms} is larger than the (second-order) shift in average adhesion. This strong *linear* perturbation of the local energy arises because the solid is stretched by a distance $u(\xi) \propto \sqrt{\xi} K/E'$ with $K = \sqrt{2 w_{\text{int}} E'}$ at an equilibrium crack tip. In valleys, the solid needs to stretch even more, increasing the elastic energy and decreasing the equivalent adhesion, while on roughness peaks, the equivalent adhesion *increases* because the solid needs to stretch less than for a perfect sphere (see also Fig. 4 of the main text). The amplitude of these energy fluctuations are given by $h_{\text{rms}}^{(1/2)}$, a generalized measure of the sharpness of peaks sensitive to larger length scales than curvatures and slopes. For self-affine roughness, this parameter is dominated either by large scales like the rms height, or by small scales like slope and curvatures, depending on the Hurst exponent (5).

D. Non-circular contact: In-plane perturbation of the elastic energy release rate

Above, we discussed the effect of out-of-plane perturbation on a perfectly circular contact. In reality, the contact shape will deviate from circularity. We now compute the energy release rate at point P on a nearly circular contact to a rough sphere,

$$G([h,a];b,\theta_\mathrm{P}) = G_\circ([h];b,a(\theta_\mathrm{P}),\theta_\mathrm{P}) + G_\parallel([a];\theta_\mathrm{P}), \tag{S28}$$

with first-order accuracy in the deviation from circularity $\delta a(\theta,\theta_\mathrm{P}) = a(\theta) - a(\theta_\mathrm{P})$, see Fig. S3. Our approximation is based on Gao and Rice's (9) first-order perturbation of the stress intensity factor at the perimeter of an initially circular external crack. Their result applies to arbitrary indenter geometries, where the stress intensity factor can vary along the perimeter (11, 59, 62), as is the case here due to surface roughness. We show that the first-order effect of the in-plane perturbation $G_\parallel$ is independent of the out-of-plane geometry, so that the contact of a rough sphere is equivalent to the contact of a smooth sphere discussed in Ref. (11) using the equivalent work-of-adhesion heterogeneity Eq. (S25). In Ref. (11), we only considered the case where $G_\circ$ is uniform over the contact perimeter, such as when $G_\circ = G_\mathrm{JKR}$. Here, we highlight the key changes required when $G_\circ$ is a function of θ.

The line elasticity emerges from the elastic coupling of the surface displacements caused by moving an initially stretched crack tip. The more the solid is stretched, the larger the elastic energy required to distort the contact line. In this section, we discuss perturbation of stress intensity factor in terms of displacements rather than pressures and introduce a displacement intensity factor (63, 64),

$$\Xi = \sqrt{8/\pi}K/E', \tag{S29}$$

in order to shorten the notation. Close to the crack tip, the geometry of the solid is described by

$$u(\xi,\theta) = \Xi(\theta)\sqrt{\xi} + \mathcal{O}(\xi^{3/2}), \tag{S30}$$

so that Ξ^2 corresponds to the diameter at the crack tip, see Fig. S3b. This length gives a

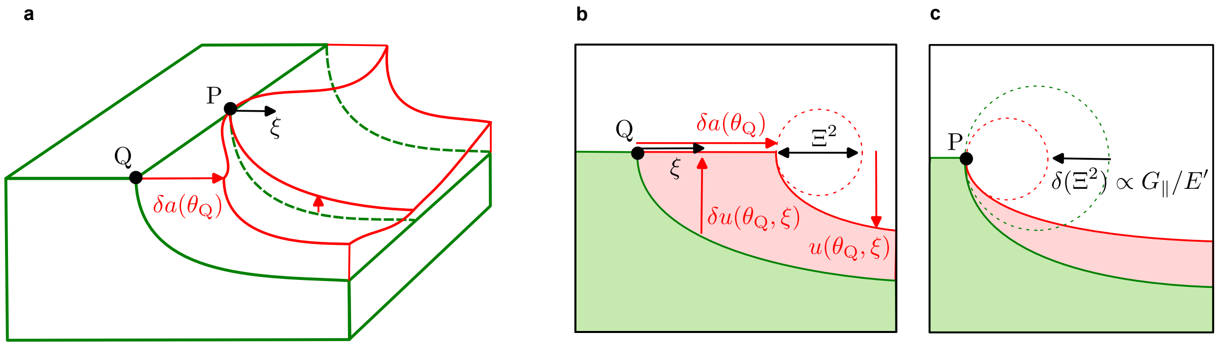

FIG. S3. **Effect of in-plane perturbations of the crack front on the energy release rate, $G_{\|}$.** (a) The green solid represents a small section of the circular reference configuration with constant radius $a(\theta_P)$ that we perturb by $\delta a(\theta_Q, \theta_P) = a(\theta_Q) - a(\theta_P)$ (red). Advancing the contact area brings the crack faces closer together even in front of the point P that we hold fixed, because of the nonlocal interaction of the surface displacements. (b) At the crack tip, the displacements $u(\xi) \simeq \Xi\sqrt{\xi}$ with displacement intensity factor Ξ, so that closing the crack faces requires displacements $\delta u(\theta_Q, \xi) = \Xi(\theta_Q)\sqrt{\xi}$. The length Ξ^2 is the out-of-plane diameter at the crack tip (red circle) and corresponds to the elastic energy release via $G \propto E'\Xi^2$. At equilibrium, this diameter is proportional to the elastoadhesive length $u_{\mathrm{ea}} = w_{\mathrm{int}}/E'$. (c) The diameter of the crack tip decreases by $\delta(\Xi^2) = 2\Xi\Xi_{\|}$ as the crack faces come together at the point P.

geometric interpretation of the energy release rate via Eq. (S5),

$$G = (\pi/16)E'\Xi^2. \tag{S31}$$

At equilibrium, $G = w_{\mathrm{int}}$, so that the diameter of the crack tip $\Xi^2_{\mathrm{eq}} = (16/\pi)u_{\mathrm{ea}}$, with the elastoadhesive length $u_{\mathrm{ea}} = w_{\mathrm{int}}/E'$ (65).

Rice explained how distorting an initially circular contact perimeter affects the energy release rate in a point P that we hold fixed, see Fig. S3a. Making use of symmetries of the elastic potential, Rice showed that the crack-face weight function describes how the surface of the solid moves as we distort the crack front within the plane. For the energy release rate, only the perturbation of crack-face displacements close to the crack tip matters. This

perturbation is described by (9)

$$\begin{aligned}\Xi_{\|}([h,a];b,\theta_P) =&\Xi([h,a];b,\theta_{\rm P}) - \Xi_\circ([h];b,a(\theta_{\rm P}),\theta_{\rm P})\\ =&-\frac{1}{2\pi}\mathrm{PV}\int_0^{2\pi} \mathrm{d}\theta_{\rm Q}\, a(\theta_{\rm P})\frac{(a(\theta_{\rm Q})-a(\theta_{\rm P}))\Xi_\circ([h];b,a(\theta_{\rm Q}),\theta_{\rm Q})}{||\vec{r_{\rm P}}-\vec{r_{\rm Q}}||^2} + \mathcal{O}([a-a(\theta_{\rm P})]^2),\end{aligned} \tag{S32}$$

where $\Xi_\circ$ is the displacement intensity factor for the perfectly circular contact including roughness. The kernel of the integral was obtained from the $\xi \to 0$ limit of the crack-face weight function of a circular external crack by Gao and Rice (9, 10, 66). Equations (S31) and (S32) combined with the results from section S1 B yield the energy release rate for the nearly circular contact to a rough sphere.

Equation (S32) captures the dominating effect of the long-ranged elastic coupling of the surface displacements on the energy release rate. We illustrate this effect in Fig. S3, where we hold $a(\theta_P)$ fixed and advance the contact area in the neighborhood, corresponding to a locally convex perturbation of the contact perimeter (Fig. S3a). Closing the adhesive neck over the surface element $\mathrm{d}\theta\, a(\theta_{\rm Q})\delta a(\theta_{\rm Q})$ requires the displacement $\delta u(\theta_{\rm Q},\xi) = \Xi(\theta_{\rm Q})\sqrt{\xi}$ (Fig. S3b), which perturbs the whole surface of the solid with amplitudes decaying with distance as $||\vec{r}_P - \vec{r}_Q||^{-2}$. We hold the crack front locally pinned in θ_P, yet this nonlocal interaction along the crack front brings the crack faces together (Fig. S3c) and thereby reduces the energy release rate $G(\theta_P)$.

We now show that within our first-order approximation, the reduction of G for convex a discussed in the previous paragraph is independent of the indenter geometry and stiffness, and corresponds to a generalized curvature, the half-fractional Laplacian $(-\Delta_s)^{1/2}a(s)$, where $s = a\,\theta$ is an arclength along the contact perimeter. The principal value integral in Eq. (S32) depends on roughness, indenter geometry, and indenter position via $\Xi_\circ$, but this coupling of the in-plane elastic response to the out-of-plane geometry is only $\mathcal{O}(\delta a^2)$. Near equilibrium, where the contact radius is such that

$$\Xi_\circ([h];b,a(\theta),\theta) + \mathcal{O}([a-a(\theta)]) = \Xi_{\rm eq} = \sqrt{\frac{16 w_{\rm int}}{\pi E'}}, \tag{S33}$$

deviations of $\Xi_\circ$ from the material property $\Xi_{\rm eq}$ manifest in Eq. (S32) through the second-

order term $\Xi_\circ \delta a$. Approximating $\Xi_\circ$ by the constant value $\Xi_{\rm eq}$ simplifies the principal value integral to

$$\Xi_{\|}([a];\theta_{\rm P}) \approx -\frac{\Xi_{\rm eq}}{2\pi}\,\mathrm{PV}\int_0^{2\pi} \mathrm{d}\theta_{\rm Q}\, a(\theta_{\rm P}) \frac{\delta a(\theta_{\rm Q})}{||\vec{r_{\rm P}} - \vec{r_{\rm Q}}||^2} = \frac{\Xi_{\rm eq}}{2}\,(-\Delta_s)^{1/2}\, a(\theta_{\rm P}). \tag{S34}$$

Here, the half-fractional Laplacian of the contact radius with respect to the arclength $\mathrm{d}s = a(\theta_{\rm P})\,\mathrm{d}\theta_{\rm P}$ is defined by

$$(-\Delta_s)^{1/2}\, a(\theta_{\rm P}) = \frac{1}{a(\theta_{\rm P})}\,(-\Delta_\theta)^{1/2}\, a(\theta_{\rm P}) = \frac{1}{a(\theta_{\rm P})} \sum_{n\in\mathbb{Z}\setminus\{0\}} |n|\; \tilde{a}_n\; e^{in\theta_{\rm P}}, \tag{S35}$$

where $\tilde{a}_n$ are the coefficients of the Fourier series

$$a(\theta) = \sum_{\mathbb{Z}} \tilde{a}_n\; e^{in\theta}. \tag{S36}$$

The wavelength of a Fourier mode is $\lambda_n = 2\pi a(\theta)/|n|$. The Fourier amplitude of $(-\Delta_s)^{1/2}\, a(\theta_{\rm P})$, $\tilde{a}_n/\lambda_n$, is the slope of the Fourier mode, but unlike slopes, the maxima and minima of the fractional Laplacian are in phase with maxima and minima of a. Hence, $(-\Delta_s)^{1/2}\, a$ can be interpreted as a generalized curvature scaling like a slope.

Equations (S33) and (S34) yield the first-order perturbation of the energy release rate

$$G_{\|}([a],\theta_{\rm P}) \approx \frac{\pi E'}{8}\Xi_{\rm eq}\Xi_{\|}([a],\theta_{\rm P}) = w_{\rm int}\,(-\Delta_s)^{1/2}\, a(\theta_{\rm P}), \tag{S37}$$

describing that the line penalizes deviations from circularity with a strength proportional to the equilibrium energy release rate $w_{\rm int}$ and a generalized curvature. This means that for a fixed jump depth $\delta a = d$, it is easier to deflect the line over a wider lateral section ℓ, $\delta G = w_{\rm int} d/\ell$, explaining why a row of several asperities can *collectively* pin the crack front while an individual asperity cannot (24).

S2. NUMERICAL IMPLEMENTATION OF THE CRACK-FRONT MODEL

Our numerical simulations use the algorithm by Rosso and Krauth (45) to solve for the equilibrium configurations (metastable states) visited by the crack front as we pull the sphere

in and out of the contact. We discretize the crack front in N collocation points at equally spaced angles θ following Ref. (11).

The surface roughness $h(x, y)$ is a Gaussian random field, where the height spectrum $\tilde{h}(q_x, q_y)$ has uncorrelated phases and random amplitudes scaling according to the power-spectral density (PSD), and defines the equivalent work-of-adhesion field via Eqs. (S13) and (S25). Equation (S13) describes the stress intensity factor for a straight crack that is rotated to be tangential to the contact circle. Note that the prefactor in Eq. (S13) is a complex number that introduces a minor phase shift between w_{loc} and h in the direction normal to the front. While this phase shift, and thereby the orientation of the crack, are important when comparing deterministically the crack-front model to the BEM, they have no effect on the power spectrum of w_{loc} and on the work of adhesion hysteresis. When the correlation length is much smaller than the contact radius, the heights decorrelate before the orientation of the crack changes substantially along the perimeter. For this reason, and because the rotation becomes computationally intractable on large grids, we generate the equivalent work-of-adhesion fields used in the main text and Sec. S4 using a constant orientation of the crack.

S3. VALIDATION OF THE CRACK-FRONT MODEL AGAINST THE BOUNDARY ELEMENT METHOD

We compare the crack-front model to a boundary element method (BEM) simulation to validate our mapping from surface roughness to an equivalent work-of-adhesion heterogeneity. The implementation of the BEM and the parameters of the simulation are similar to Ref. (11), where we validated the crack-front model for spheres with heterogeneous work of adhesion. In the BEM simulation we perform here, the sphere is rough and the work of adhesion is uniform. The surfaces interact with a cubic cohesive law with a hard-wall repulsion. Our implementation of the BEM is described in detail in Ref. (11) and is based on (4, 35, 67–70).

Figure S4 shows a BEM and a crack-front simulation on random roughness with $e_{\mathrm{el}}/w_{\mathrm{int}} \simeq 0.03$ and a power spectrum that is flat for wavelengths above the correlation length $\lambda_{\mathrm{r}} = 0.2$ and 0 below. The force-penetration curves computed with the BEM and the crack-front model nearly overlap and contact perimeters agree well, confirming that the contact of

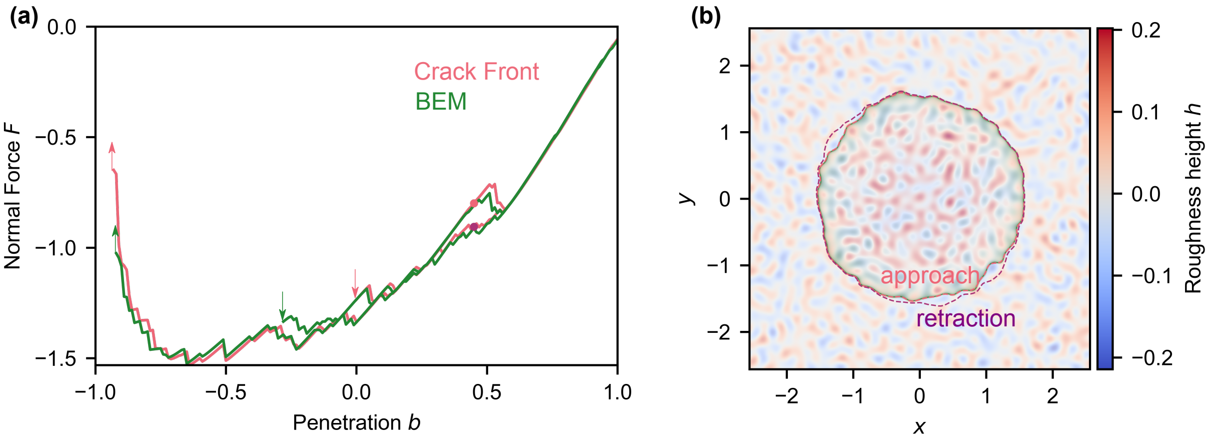

FIG. S4. **Validation of the crack-front model with the boundary-element method (BEM). (a)** Force-penetration curves from a BEM and a crack-front simulation on the random roughness shown in panel (b). The arrows indicate the jump-into-contact and the jump-out-of-contact instabilities. **(b)** Contact areas at the penetration $b = 0.45$, indicated by the dots in the force-penetration curve, on top of the surface topography. The tensile pressures of the contact mechanics simulation during approach are shown in green, so that the perimeter of the contact is indicated by the darkest green pixels. The dashed lines represent the contact perimeter calculated with the crack-front model during approach (pink) and retraction (purple). The BEM simulation was discretized on a 1024×1024 grid with pixel size 0.005. The roughness is a random Gaussian field with a flat power spectrum at wavelengths above the cutoff wavelength $\lambda_\mathrm{r} = 0.2$ and 0 below. The interaction is a cubic polynomial with a cutoff distance $g_c = 0.24$, corresponding to a cohesive zone size $\xi_\mathrm{coz} = (\pi/36) g_c^2/u_\mathrm{ea} \simeq 0.012$, with the elastoadhesive length $u_\mathrm{ea} = w_\mathrm{int}/E'$. In both simulations, we increased the penetration b in steps of 0.01 until the maximum penetration $b_\mathrm{max} = 1$ was reached and then decreased it until pull off. The results are nondimensionalized following the conventions of Refs. (47, 48).

rough spheres is equivalent to the pinning of a crack by the work-of-adhesion heterogeneity w_loc mapped using equation (S25). Note that in the BEM, the jump-into-contact instability occurs too early because of the finite interaction range (11, 71–73). This particular event converges slowly with interaction range, while the remainder of the force-penetration curve, including depinning instabilities, is well converged. Other discrepancies in the force-penetration curves are due to the linearization in the crack-front model.

S4. HYSTERESIS ON RANDOM ROUGHNESS: CRACK-FRONT SIMULATIONS

We verify our theoretical prediction for the apparent work of adhesion (Main Text Eq. (6))

$$w_{\substack{\text{retr}\\ \text{appr}}} = w_{\text{int}} - e_{\text{el}} \pm k e_{\text{el}}, \tag{S38}$$

using crack-front simulations on self-affine roughness with varying power spectra, and extract the numerical factor $k \approx 3$ from these results. We show in Suppl. Sec. S1B and S1C that self-affine surface roughness maps to an equivalent work-of-adhesion field with power-law correlation via the integral transform Eq. (S13). The variance of this work-of-adhesion heterogeneity $w_{\text{rms}}^2 = 4e_{\text{el}}w_{\text{int}}$, with e_{el} the elastic energy required to conform to the surface roughness.

Figure S5 shows the work of adhesion hysteresis $w_{\text{retr}} - w_{\text{appr}}$ as a function the amplitude of disorder $(w_{\text{rms}}/w_{\text{int}})^2 = 4e_{\text{el}}/w_{\text{int}}$. The dashed line represents the prediction Eq. (S38) using $k \approx 3$ which we fitted to the results. For $e_{\text{el}}/w_{\text{int}} \gtrsim 0.01$, the work of adhesion hysteresis in our numerical simulations (symbols) overlaps with the theoretically predicted scaling (dashed line) and is independent of the shape of the power spectrum. Below a critical value of e_{el}, the hysteresis disappears because of the finite size of the contact (13, 14, 74). This onset of hysteresis depends on the shape of the power-spectral density: for roughness with a short correlation length (purple triangles), the contact process starts to dissipate energy at smaller e_{el} than for a longer correlation length (pink crosses).

The scaling of the hysteresis with w_{rms}^2 was theoretically predicted and numerically verified on random fields with short-ranged correlation (12, 14, 27), similar to our roughness with flat PSD represented by the green squares. Here we consider isotropic self-affine roughness leading to work-of-adhesion fields with long-ranged power-law correlations. Démery et al. (27) theoretically predicted that for isotropic fields, the relationship between hysteresis and w_{rms} remains unaffected by these power-law correlations. They derived this result by analytically solving a small disorder expansion of the equation of motion of the elastic line. Our numerical simulations further confirm that Eq. (S38) remains valid for isotropic self-affine roughness.

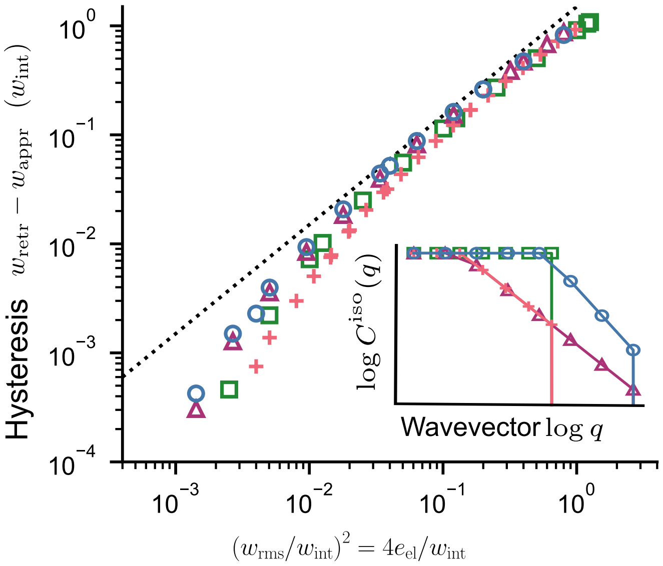

FIG. S5. **Dependence of the hysteresis on the elastic energy for fully conformal contact, $e_{\rm el}$.** Crack-front simulations on random fields of adhesion $W(x, y)$ show that the hysteresis in apparent work of adhesion $w_{\rm retr} - w_{\rm appr} \propto w_{\rm rms}^2$ (dashed line). We show in Suppl. Sec. S1B and S1C that surface roughness with elastic energy for fully conformal contact $e_{\rm el}$ is equivalent to a work-of-adhesion heterogeneity with variance $w_{\rm rms}^2 = 4e_{\rm el}w_{\rm int}$. The work-of-adhesion fields used here correspond to randomly rough surfaces with different shapes of the power spectra represented in the inset. We used a flat PSD with short-wavelength cutoff $\lambda_s = 0.005$ (green squares), and three different self-affine PSDs parameterized by the rolloff wavelength λ_r, the short-wavelength cutoff λ_s and the Hurst exponent H. The self-affine PSDs scale with $|q|^{-2-2H}$ for wavelengths between λ_s and λ_r. The blue circles correspond to $\lambda_s = 0.000625, \lambda_r = 0.01, H = 0.8$; the purple triangles to $\lambda_s = 0.000625, \lambda_r = 0.1, H = 0.3$; and the pink crosses to $\lambda_s = 0.005, \lambda_r = 0.1, H = 0.3$. The force-area curve for the blue circle at $e_{\rm el}/w_{\rm int} = 0.05$ is shown in Main Text Fig. 3C.

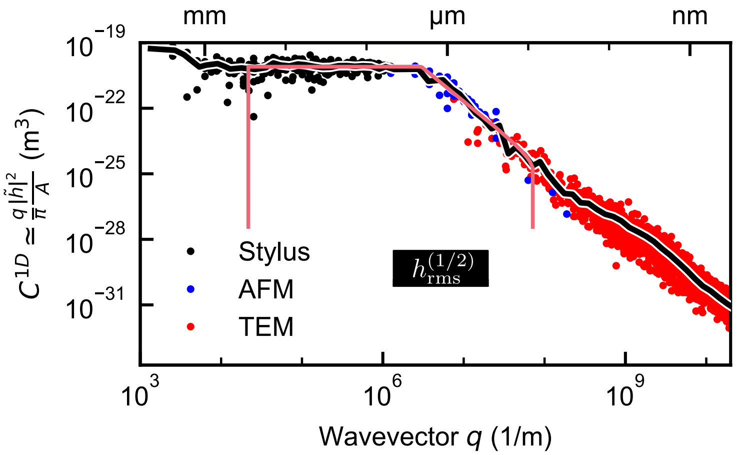

FIG. S6. **One-dimensional power-spectral density of the nanocrystalline rough surface and of the synthetic surface used in the simulation shown in main-text Fig. 5a.** We fit an ideal self-affine PSD with Hurst exponent $H = 1$ to the experimental data (light red line). The rolloff wavelength is $2.1\,\mu$m and we chose the amplitude of the PSD to match the elastic energy for fully conformal contact, giving the amplitude of the 2D PSD in the rolloff region is $8.4 \cdot 10^{-27}$ m. The vertical lines show the length scales included in our simulation.

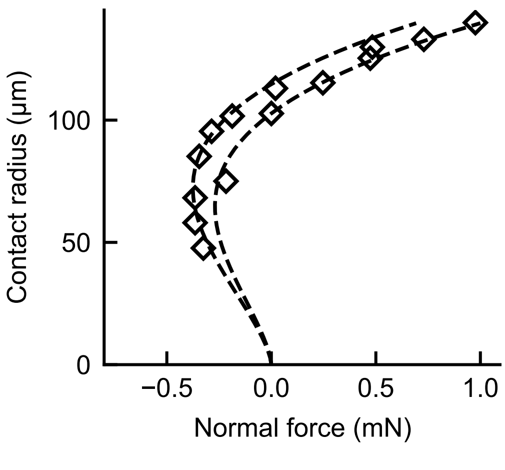

FIG. S7. **Contact radius and normal force during approach and retraction of a rubber sphere against an atomically flat silicon wafer covered with octadecyltrichlorosilane (OTS).** The data shows that the hysteresis is low in the absence of surface roughness. The dashed lines are fitted JKR curves with work of approach $\simeq 47\,\mathrm{mJ/m^2}$ and work of retraction of $\simeq 68\,\mathrm{mJ/m^2}$, corresponding to a hysteresis of $\simeq 21\,\mathrm{mJ/m^2}$.

Movie S1 *In situ* video recording of the contact area in the indentation-retraction experiment shown in Fig. 1 and Fig. 5. The contact area appears as a bright patch. We speed up the frame rate by a factor of 10 compared to the original recording.

Movie S2 Video comparing the extracted contact perimeters (dashed red line) and the original grayscale image from the experiment. In Fig. 5b, we only show the bottom right section of the perimeter, where the brightness contrast between contact and non-contact area is good so that the extracted line follows well the contour of the contact area.

REFERENCES AND NOTES

1. J. N. Israelachvili, *Intermolecular and Surface Forces* (Academic Press, 1991).
2. K. L. Johnson, K. Kendall, A. D. Roberts, Surface energy and the contact of elastic solids, *Proc. R. Soc. London, Ser. A* **324**, 301– 313 (1971).
3. B. N. J. Persson, O. Albohr, U. Tartaglino, A. I. Volokitin, E. Tosatti, On the nature of surface roughness with application to contact mechanics, sealing, rubber friction and adhesion, *J. Phys. Condens. Matter* **17**, R1 (2005).
4. L. Pastewka, M. O. Robbins, Contact between rough surfaces and a criterion for macroscopic adhesion, *Proc. Natl. Acad. Sci. U.S.A.* **111**, 3298–3303 (2014).
5. B. N. J. Persson, E. Tosatti, The effect of surface roughness on the adhesion of elastic solids, *J. Chem. Phys.* **115**, 5597–5610 (2001).
6. M. K. Chaudhury, G. M. Whitesides, Direct measurement of interfacial interactions between semispherical lenses and flat sheets of poly(dimethylsiloxane) and their chemical derivatives, *Langmuir* **7**, 1013–1025 (1991).
7. S. Dalvi, A. Gujrati, S. R. Khanal, L. Pastewka, A. Dhinojwala, T. D. B. Jacobs, Linking energy loss in soft adhesion to surface roughness, *Proc. Natl. Acad. Sci. U.S.A.* **116**, 25484–25490 (2019).
8. Y. L. Chen, C. A. Helm, J. N. Israelachvili, Molecular mechanisms associated with adhesion and contact angle hysteresis of monolayer surfaces, *J. Phys. Chem.* **95**, 10736–10747 (1991).
9. H. Gao, J. R. Rice, Nearly circular connections of elastic half spaces, *J. Appl. Mech.* **54**, 627–634 (1987).
10. J. R. Rice, Weight function theory for three-dimensional elastic crack analysis, in *Fracture Mechanics: Perspectives and Directions (Twentieth Symposium)*, R. Wei, R. Gangloff, Eds. (American Society for Testing and Materials, 1989), pp. 29–57.
11. A. Sanner, L. Pastewka, Crack-front model for adhesion of soft elastic spheres with chemical heterogeneity, *J. Mech. Phys. Solids* **160**, 104781 (2022).
12. A. I. Larkin, Y. N. Ovchinnikov, Pinning in type II superconductors, *J. Low. Temp. Phys.* **34**, 409–428 (1979).
13. M. O. Robbins, J. F. Joanny, Contact angle hysteresis on random surfaces, *Europhys. Lett.* **3**, 729– 735 (1987).
14. V. Démery, A. Rosso, L. Ponson, From microstructural features to effective toughness in disordered brittle solids, *Europhys. Lett.* **105**, 34003 (2014).

15. A. Gujrati, S. R. Khanal, L. Pastewka, T. D. B. Jacobs, Combining TEM, AFM, and profilometry for quantitative topography characterization across all scales, *ACS Appl. Mater. Interf.* **10**, 29169–29178 (2018).
16. A. Gujrati, *et al.*, Comprehensive topography characterization of polycrystalline diamond coatings, *Surf. Topogr. Metrol. Prop.* **9**, 014003 (2021).
17. A. A. Griffith, G. I. Taylor, *VI. The phenomena of rupture and flow in solids*, *Philos. Trans. R. Soc. London, Ser. A* **221**, 163– 198 (1921).
18. P. R. Guduru, Detachment of a rigid solid from an elastic wavy surface: Theory, *J. Mech. Phys. Solids* **55**, 445– 472 (2007).
19. H. Kesari, J. C. Doll, B. L. Pruitt, W. Cai, A. J. Lew, Role of surface roughness in hysteresis during adhesive elastic contact, *Philos. Mag. Lett.* **90**, 891–902 (2010).
20. H. Kesari, A. J. Lew, Effective macroscopic adhesive contact behavior induced by small surface roughness, *J. Mech. Phys. Solids* **59**, 2488–2510 (2011).
21. V. L. Popov, Adhesion hysteresis due to chemical heterogeneity, in *Multiscale Biomechanics and Tribology of Inorganic and Organic Systems*, G.-P. Ostermeyer, V. L. Popov, E. V. Shilko, O. S. Vasiljeva, Eds., Springer Tracts in Mechanical Engineering (Springer International Publishing, 2021), pp. 473–483.
22. J. R. Rice, First-order variation in elastic fields due to variation in location of a planar crack front, *J. Appl. Mech.* **52**, 571–579 (1985).
23. J. M. Monti, M. O. Robbins, Sliding friction of amorphous asperities on crystalline substrates: Scaling with contact radius and substrate thickness, *ACS Nano* **14**, 16997–17003 (2020).
24. Y. Imry, S.-K. Ma, Random-field instability of the ordered state of continuous symmetry, *Phys. Rev. Lett.* **35**, 1399– 1401 (1975).
25. J. F. Joanny, P. G. de Gennes, A model for contact angle hysteresis, *J. Chem. Phys.* **81**, 552–562 (1984).
26. S. Patinet, D. Vandembroucq, S. Roux, Quantitative prediction of effective toughness at random heterogeneous interfaces, *Phys. Rev. Lett.* **110**, 165507 (2013).
27. V. Démery, V. Lecomte, A. Rosso, The effect of disorder geometry on the critical force in disordered elastic systems, *J. Stat. Mech.* **2014**, P03009 (2014).
28. T. D. B. Jacobs, T. Junge, L. Pastewka, Quantitative characterization of surface topography using spectral analysis, *Surf. Topogr. Metrol. Prop.* **5**, 013001 (2017).

29. I. A. Lyashenko, R. Pohrt, Adhesion between rigid indenter and soft rubber layer: Influence of roughness, *Front. Mech. Eng.* **6**, 49 (2020).
30. B. N. J. Persson, E. A. Brener, Crack propagation in viscoelastic solids, *Physical Review E* **71**, 036123 (2005).
31. M. H. Müser, B. N. J. Persson, Crack and pull-off dynamics of adhesive, viscoelastic solids, *Europhys. Lett.* **137**, 36004 (2022).
32. B. N. J. Persson, Adhesion between elastic bodies with randomly rough surfaces, *Phys. Rev. Lett.* **89**, 245502 (2002).
33. K. N. G. Fuller, D. Tabor, *The effect of surface roughness on the adhesion of elastic solids*, *Proc. R. Soc. London, Ser. A* **345**, 327–342 (1975).
34. N. Mulakaluri, B. N. J. Persson, Adhesion between elastic solids with randomly rough surfaces: Comparison of analytical theory with molecular-dynamics simulations, *Europhys. Lett.* **96**, 66003 (2011).
35. L. Pastewka, M. O. Robbins, Contact area of rough spheres: Large scale simulations and simple scaling laws, *Appl. Phys. Lett.* **108**, 221601 (2016).
36. A. Wang, M. H. Müser, Is there more than one stickiness criterion?, *Friction* **11**, 1027–1039 (2023).
37. S. Medina, D. Dini, A numerical model for the deterministic analysis of adhesive rough contacts down to the nano-scale, *Int. J. Solids Struct.* **51**, 2620 (2014), 2632.
38. V. L. Popov, R. Pohrt, Q. Li, Strength of adhesive contacts: Influence of contact geometry and material gradients, *Friction* **5**, 308–325 (2017).
39. V. L. Popov, Q. Li, I. A. Lyashenko, R. Pohrt, Adhesion and friction in hard and soft contacts: Theory and experiment, *Friction* **9**, 1688–1706 (2021).
40. K. L. Johnson, *Contact Mechanics* (Cambridge Univ. Press, 2012).
41. B. B. Mandelbrot, D. E. Passoja, A. J. Paullay, Fractal character of fracture surfaces of metals, *Nature* **308**, 721– 722 (1984).
42. T. Candela, F. Renard, Y. Klinger, K. Mair, J. Schmittbuhl, E. E. Brodsky, Roughness of fault surfaces over nine decades of length scales, *J. Geophys. Res. – Sol. Ea.* **117**, B08409 (2012).
43. B. N. J. Persson, On the fractal dimension of rough surfaces, *Tribol. Lett.* **54**, 99–106 (2014).
44. G. A. D. Briggs, B. J. Briscoe, Effect of surface roughness on rolling friction and adhesion between elastic solids, *Nature* **260**, 313–315 (1976).

45. A. Rosso, W. Krauth, Roughness at the depinning threshold for a long-range elastic string, *Phys. Rev. E* **65**, 025101 (2002).
46. A. Gujrati, Nanocrystalline diamond (version 2). NCD surface topography measured using TEM, AFM, and stylus profilometry; https://doi.org/10.57703/ce-9npwd (2023).
47. E. Barthel, Adhesive elastic contacts: JKR and more, *J. Phys. D Appl. Phys.* **41**, 163001 (2008).
48. D. Maugis, *Contact, Adhesion and Rupture of Elastic Solids* (Springer, 2010).
49. P. M. Anderson, J. R. Rice, The stress field and energy of a three-dimensional dislocation loop at a crack tip, *J. Mech. Phys. Solids* **35**, 743–769 (1987).
50. H. Hertz, Über die Berührung fester elastischer Körper, *J. Reine Angew. Math.* **92**, 156 (1881).
51. I. N. Sneddon, Boussinesq's problem for a flat-ended cylinder, *Math. Proc. Cambridge Philos. Soc.* **42**, 29–39 (1946).
52. G. R. Irwin, Analysis of stresses and strains near the end of a crack transversing a plate, *J. Appl. Mech.* **24**, 361–364 (1957).
53. V. M. Muller, V. S. Yushchenko, B. V. Derjaguin, On the influence of molecular forces on the deformation of an elastic sphere and its sticking to a rigid plane, *J. Colloid Interface Sci.* **77**, 91–101 (1980).
54. M. H. Müser, Single-asperity contact mechanics with positive and negative work of adhesion: Influence of finite-range interactions and a continuum description for the squeeze-out of wetting fluids, *Beilstein J. Nanotechnol.* **5**, 419–437 (2014).
55. A. T. Zehnder, *Fracture Mechanics*, no. 62, in Lecture Notes in Applied and Computational Mechanics (Springer Science+Business Media, 2012).
56. H. M. Westergaard, General solution of the problem of elastostatics of an n-dimensional homogeneous isotropic solid in an n-dimensional space, *Bull. Am. Math. Soc.* **41**, 695–699 (1935).
57. H. Tada, P. C. Paris, G. R. Irwin, *The Stress Analysis Of Cracks Handbook* (ASME Press, ed. 3, 2000).
58. M. Abramowitz, I. A. Stegun, *Handbook of Mathematical Functions with Formulas, Graphs, and Mathematical Tables*, vol. 55 (U.S. Government Printing Office, 1964).
59. N. M. Borodachev, Contact problem for an elastic half-space with a near-circular contact area, *Sov. Appl. Mech.* **27**, 118–123 (1991).
60. V. I. Fabrikant, Stress intensity factors and displacements in elastic contact and crack problems, *J. Eng. Mech.* **124**, 991–999 (1998).

61. I. Argatov, A comparison of general solutions to the non-axisymmetric frictionless contact problem with a circular area of contact: When the symmetry does not matter, *Symmetry* **14**, 1083 (2022).
62. J. R. Rice, Three-dimensional elastic crack tip interactions with transformation strains and dislocations, *Int. J. Solids Struct.* **21**, 781–791 (1985).
63. M. L. Williams, On the stress distribution at the base of a stationary crack, *J. Appl. Mech.* **24**, 109–114 (1957).
64. R. Hartranft, G. Sih, The use of eigenfunction expansions in the general solution of three-dimensional crack problems, *Indiana Univ. Math. J.* **19**, 123–138 (1969).
65. C. Creton, M. Ciccotti, Fracture and adhesion of soft materials: A review, *Rep. Prog. Phys.* **79**, 046601 (2016).
66. G. M. L. Gladwell, Ed., *Contact Problems*, vol. **155** of *Solid Mechanics and Its Applications* (Springer Netherlands, 2008).
67. R. W. Hockney, The potential calculation and some applications, in *Methods in Computational Physics,* vol. 9, B. A. Alder, S. Fernbach, M. Rotenberg, Eds. (Academic Press, 1970), pp. 135–211.
68. R. H. Byrd, P. Lu, J. Nocedal, C. Zhu, A limited memory algorithm for bound constrained optimization, *SIAM J. Sci. Comput.* **16**, 1190– 1208 (1995).
69. H. M. Stanley, T. Kato, An FFT-based method for rough surface contact, *J. Tribol.* **119**, 481– 485 (1997).
70. C. Campañá, M. H. Müser, Practical Green's function approach to the simulation of elastic semi-infinite solids, *Phys. Rev. B* **74**, 075420 (2006).
71. J.-J. Wu, The jump-to-contact distance in atomic force microscopy measurement, *J. Adhes.* **86**, 1071–1085 (2010).
72. M. Ciavarella, J. A. Greenwood, J. R. Barber, Effect of Tabor parameter on hysteresis losses during adhesive contact, *J. Mech. Phys. Solids* **98**, 236– 244 (2017).
73. A. Wang, Y. Zhou, M. H. Müser, Modeling adhesive hysteresis, *Lubricants* **9**, 17 (2021).
74. A. Tanguy, T. Vettorel, From weak to strong pinning I: A finite size study, *Eur. Phys. J. B.* **38**, 71– 82 (2004).
